# More Than a Haematoma: A Case of Aplastic Anemia

**DOI:** 10.7759/cureus.75689

**Published:** 2024-12-14

**Authors:** Gaspar Fernandes, Tiago Ruivo, Óscar Ramos

**Affiliations:** 1 Family Medicine, Family Health Unit (USF) Almedina, Local Health Unit of Trás-os-Montes and Alto Douro (ULSTMAD), Lamego, PRT

**Keywords:** acquired aplastic anemia, bone marrow failure, bruising, ecchymosis, laboratory findings of pancytopenia, severe aplastic anemia

## Abstract

Easy bruising and ecchymosis are common symptoms in clinical practice, yet distinguishing benign from clinically significant cases can be challenging. We report the case of a 46-year-old woman who presented in December 2023 with easy bruising and increased menstrual flow, revealing new-onset pancytopenia in laboratory tests. Initially diagnosed with Acute Myeloid Leukemia inversion (inv) (16), subsequent results were inconclusive, leading to a diagnosis of Paroxysmal Nocturnal Hemoglobinuria (PNH). Following clinical and analytical deterioration, a repeat bone marrow study in March 2024 identified Aplastic Anemia (AA). When evaluating easy bruising and ecchymosis, a thorough history and physical examination are essential, sometimes requiring further investigation and referral. AA is a rare and heterogeneous disease that requires excluding other causes of bone marrow failure for proper diagnosis. Its severity and the patient’s age determine treatment and prognosis. This case underscores the importance of investigating common symptoms that may signal serious underlying conditions, as well as the complexities involved in diagnosing AA.

## Introduction

Easy bruising/ecchymosis is a common symptom encountered in both primary care (PC) and hematology consultations. Differentiating between normal and clinically significant bruising is often challenging, especially when other specific signs or symptoms are absent [1,2]. 

This case presents the diagnostic dilemma of easy bruising in a patient with a positive family history of a hematologic disorder and abnormal blood test results. Despite initial concerns about more common hematologic conditions, a thorough investigation ultimately led to the diagnosis of Aplastic Anemia (AA), highlighting the complexities and challenges in diagnosing this rare and often subtle condition.

## Case presentation

A 46-year-old female patient presented to our PC consultation facility on December 15th, 2023, with a six-month history of easy bruising on the trunk and limbs without trauma, along with increased menstrual flow. She has a history of submucosal uterine leiomyoma excised by hysteroscopy in July 2020, with no other relevant medical-surgical history, no regular medication, or known drug allergies. Family history included a sister diagnosed with paroxysmal nocturnal hemoglobinuria (PNH), with no other relevant family history. She reported pruritic skin lesions in October 2023, requiring oral corticosteroid therapy with prednisolone without further complications during this period. She denied recent use of other drugs, such as non-steroidal anti-inflammatory drugs (NSAIDs) or metamizole; she also denied other recent infectious events. Physical examination was unremarkable. A complete blood count (CBC) and prothrombin time (PT) were requested.

On December 19th, 2023, the patient came back with the results, revealing hemoglobin (Hb) 9.8 g/dL; white blood count (WBC) 4200/µL, with reversal of neutrophil-to-lymphocyte ratio (neutrophils 25.8%, lymphocytes 66.5%); platelets (Plt) 31000/µL, and a normal PT. The patient was then asked to repeat the laboratory tests on the same day. The results were: Hb 9.4 g/dL; WBC 4000/µL (neutrophils 36.1%, lymphocytes 58.1%); Plt 17440/µL; reticulocytes 56.04/µL; slight elevation of C-reactive protein (8.3 mg/L) and erythrocyte sedimentation rate (28 mm/h); normal liver and renal function and normal clotting times.

The patient was referred to the emergency department on the same day at the local hospital, where additional blood tests at admission included a peripheral blood smear, showing dysplasia in neutrophil segmentation, no observable blasts, and moderate macrocytosis. She was admitted to the hematology department to investigate new-onset pancytopenia. The results of other diagnostic tests performed during hospitalization are shown in Tables 1, 2.

**Table 1:** Additional blood tests conducted during hospitalization in the hematology department of local hospital Ab – antibody; Ag – antigen; anti-HBc – hepatitis B core antibody; Anti-HBs – hepatitis B surface antibody; Anti-HCV – hepatitis C antibody; APTT – activated partial thromboplastin time; CMV – cytomegalovirus; EBV – Epstein-Barr virus; ENAS – extractable nuclear antibodies; HBsAg – hepatitis B surface antigen; HCV – hepatitis C virus; HIV – human immunodeficiency virus; IgG – Immunoglobulin G; IgM – Immunoglobulin M; LDH – Lactate dehydrogenase; VCA – virus capsid antigen.

Parameter	Value	Interpretation
Albumin	4.0 g/dL	Normal
Creatinine	0.80 mg/dL	Normal
Total Bilirubin	0.4 md/dL	Normal
LDH	196 U/L	Normal
Ferritin	82.0 ng/mL	Normal
Folic Acid	11.3 ng/mL	Normal
Vitamin B12	232.0 pg/mL	Normal
Direct Antiglobulin Test	-	Negative
Indirect Antiglobulin Test	-	Negative
Anti-Neutrophil Cytoplasmic Ab	<1:20	Negative
Anti-Cardiolipin IgG	0.7 GPL U/mL	Negative
Anti-Cardiolipin IgM	1.7 MPL U/mL	Negative
Beta2-Glycoprotein 1 IgG	<0.6 U/mL	Negative
Beta2-Glycoprotein 1 IgM	<0.9 U/mL	Negative
ENAS Screening	-	Negative
HBsAg	0.40	Negative
Total anti-HBc	2.320	Negative
Anti-HBs	2.00 UI/L	Negative
Anti-HCV	0.10	Negative
HIV 1 and 2 Ab	0.20	Negative
CMV IgM Ab	0.070	Negative
CMV IgG Ab	170.5 AU/mL	Reactive (immune)
VCA IgM (EBV)	0.01	Negative
VCA IgG (EBV)	51.99	Reactive (immune)
Erythropoietin	334.0 mUI/mL	High
Lupus Anticoagulant	1.1	Negative
Fibrinogen	369.0 mg/dL	Normal
Prothrombin Time	13.5 seg	Normal
APTT	27.3 seg	Normal

**Table 2:** Results of bone marrow study conducted during hospitalization in the hematology department of local hospital inv – inversion; BM – bone marrow; CBFB – core binding factor beta; FISH – fluorescence in situ hybridization.

Parameter	Interpretation
BM (Myelogram)	Bone marrow was well represented with apparent erythrocyte macrocytosis. No increase in blast cells was observed. Rare megakaryocytes are present in smears with rare platelets.
BM (Genetics)	Karyotype: 46,XX,inv(16)(p13.1q22)(?)(2)/47,XX,+mar(6)/46,XX(12). Two metaphases were observed with suspected chromosome 16 inversion. A small chromosome (marker) was detected in six metaphases but could not be classified.
	FISH Study: 11% of nuclei showed a single fusion signal for the CBFB probe, indicative of inversion or translocation.

Given these findings, a diagnosis of Acute Myeloid Leukemia (AML) inversion (inv) (16)/t (16;16) was made, and the patient was transferred to the reference hospital center on December 26th, 2023. Due to diagnostic uncertainty, a repeat bone marrow study was conducted on December 27th, 2023, with provisional results showing a mildly hypocellular marrow with granulocytic dysplasia but without blast excess; immunophenotyping with CD34 (+) 0.5% and no monoclonality in B lymphocytes; CBFB::MYH11 types A, D, and E transcripts negative.

Therefore, the two bone marrow aspirate samples were concordant in the absence of blast excess; however, results were discrepant for the diagnosis, given that FISH and karyotype were positive in the sample collected at the local hospital, but molecular genetics was negative at the reference center for typical AML inv (16)/t (16;16) alterations.

Given these findings and the patient’s clinical and analytical stability, she was discharged on January 2nd, 2024, pending definitive outpatient results, presented in Table 3, with transfusional support at the local hospital and a remote consultation at the reference center for further case management.

**Table 3:** Final results of the bone marrow study conducted at reference hospital center on 12/27/2023 AML – acute myeloid leukemia; BM – bone marrow; MDS – myelodysplastic syndrome.

Parameter	Interpretation
BM (myelogram)	The findings of the current myelogram are nonspecific, but considering the signs of dysplasia and hypocellularity, and depending on the presence of clonal markers, they may align with the context of a hypoplastic MDS or bone marrow hypoplasia. There is no excess of blasts to support an AML diagnosis based on morphology. Cellularity should be confirmed by biopsy due to the possibility of aspirate hemodilution. Bone marrow lymphocytosis should also be confirmed and studied by other techniques.
BM (histology)	Histological examination of the bone fragment shows mildly/moderately hypocellular bone marrow, trilineage, with a decreased myeloid/erythroid ratio, without significant alterations in myeloid and megakaryocytic maturation. In the erythroid lineage, slight left shift/megaloblastoid maturation is observed. Tiny lymphoid aggregates and interstitial mast cells are present. In the bone marrow stroma, the reticulin network is slightly diffusely increased (MF1), and iron deposits are not apparent. Immunocytochemistry study (I24/42) confirmed the relative proportions of the myeloid and erythroid lineages (positive for myeloperoxidase and glycophorin, respectively). No immature hematopoietic cells positive for CD34 or CD117/c-kit were identified.

In summary, the bone marrow study at the reference center revealed a hypoplastic bone marrow with mild dyserythropoiesis, without features supporting a diagnosis of myelodysplastic syndrome (MDS), acute leukemia, or other neoplastic processes.

The patient resumed hematology follow-up at the local hospital, where she underwent high-sensitivity flow cytometry screening for PNH, revealing a small PNH clone in neutrophils, monocytes, and erythrocytes, thus supporting the diagnosis. Throughout January and February 2024, the patient had weekly CBCs showing progressive thrombocytopenia, complaints of fatigue, and reappearance of multiple small ecchymoses. These findings prompted a repeat bone marrow aspiration on March 6th, 2024, with results presented in Table 4.

**Table 4:** Results of bone marrow study conducted at local hospital on 03/06/2024 inv – inversion; BM – bone marrow; FISH – fluorescence in situ hybridization; MDS – myelodysplastic syndrome.

Parameter	Interpretation
BM (myelogram)	Diluted smears with peripheral blood, with clots and without megakaryocytes; evaluation not possible.
BM (genetics)	Karyotype: 47,XX,+mar(6)/46,XX(14)46,XX,inv(16)(p13.1q22)(?)(2)/47,XX,+mar(6)/46,XX(12). In six metaphases, a small chromosome of unknown origin ("marker") was observed.
	FISH study: MDS and Inv(16) negative.
BM (histology)	Hypocellular bone marrow with morphological features compatible with Acquired Aplastic Anemia.

**Figure 1:**
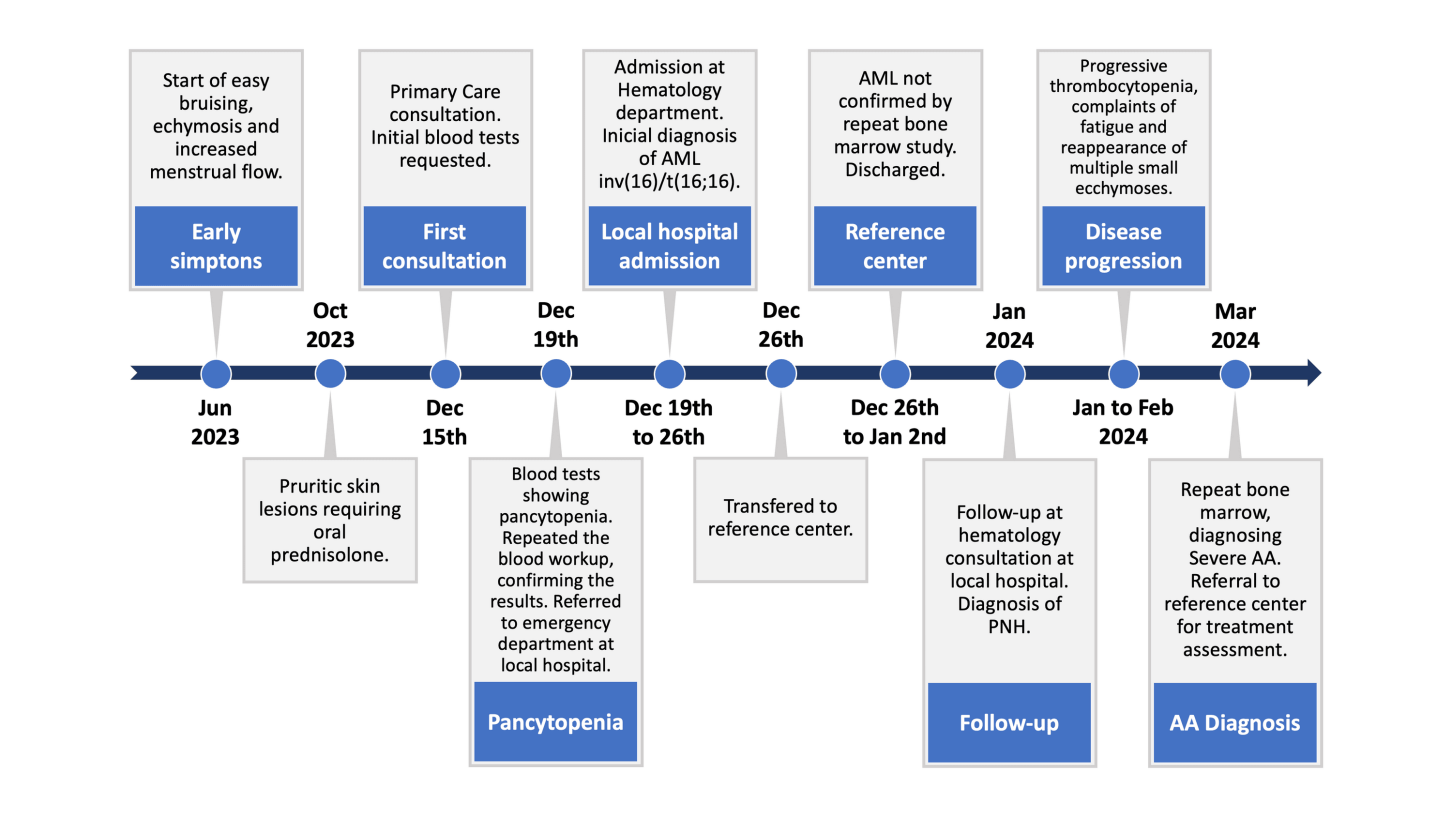
Timeline of the most relevant events AA – aplastic anemia; AML – acute myeloid leukemia; inv – inversion; PNH – paroxysmal nocturnal hemoglobinuria.

Thus, a diagnosis of severe aplastic anemia (neutrophils 2.14x10³/µL) was made, and she was referred back to the reference center for allogeneic transplantation, currently undergoing pre-transplant assessment, with regular transfusion support needed due to worsening cytopenias. Figure 1 presents a timeline summarising the most important events in chronological order.

## Discussion

The most frequent etiologies of ecchymosis can be grouped into vascular and surrounding tissue dysfunction, platelet disorders, and coagulation abnormalities [1]. The various pathologies are listed in Table 5.

**Table 5:** Causes of easy bruising in adults NSAIDs – nonsteroidal anti-inflammatory drugs. Adapted from Harrison et al. [2].

Common	Rare
Senile purpura	Hemophilia and rare coagulation factor deficiencies (I, II, V, VII, XI)
Drugs (antiplatelet agents, anticoagulants, NSAIDs)	Acquired von Willebrand disease or hemophilia
Chronic alcoholism and/or liver cirrhosis	Connective tissue diseases (e.g., Ehlers-Danlos Syndrome)
von Willebrand disease	Endocrine diseases: Cushing syndrome and hypothyroidism
Simple purpura	Hematologic neoplasms
Vitamin C and/or vitamin K deficiency	
Vasculitis	
Gastrointestinal tract disease	

A thorough clinical history (focusing on age, chronic medication, comorbidities, nutritional status, and family history of bleeding disorders) and physical examination are essential to assess bruise severity and aid in identifying underlying mechanisms, as outlined in Table 6 [1,2]. 

**Table 6:** Signs and symptoms indicative of bruise etiology Adapted from Kraut and Fenton [1] and Harrison et al. [2] studies.

Signs/Symptoms	Probable Etiology
Spontaneous bruise or bruise secondary to minor trauma	Hemorrhagic diathesis
Bruise restricted to limbs	Trauma
Bruise located on the trunk or other body areas	Hemorrhagic diathesis
Five or more bruises larger than 1 cm	Hemorrhagic diathesis
Simultaneous occurrence of petechiae and purpura	Hemorrhagic diathesis
Thin hair, brittle nails, and cachexia	Malnutrition
Periodontal inflammation	Vitamin C deficiency
Jaundice, ascites, and hepatomegaly	Chronic liver disease
Hemarthroses or joint hypermobility	Connective tissue disease
Lymphadenopathy	Infection, connective tissue disease, or lymphoid neoplasia

In many cases, other signs and symptoms associated with easy bruising/ecchymosis may not be easily identifiable, requiring further investigation to determine its etiology [2]. For this purpose, an analytical study with the following parameters should be requested, when appropriate: complete blood count, blood smear, coagulation study, creatinine, and liver function parameters [2,3]. 

Patients with easy bruising presenting with any of the following conditions should be referred to a hematologist: persistent, significant bruising (five or more bruises larger than 1 cm in diameter) without associated trauma; personal or family history of abnormal bleeding following surgeries or injuries; bleeding at other sites (such as hemarthrosis, gingival bleeding, recurrent epistaxis); laboratory abnormalities indicative of a bleeding diathesis; normal blood results but high suspicion of a bleeding disorder (based on personal and family history, and physical exam); the patient is due to have planned elective surgery or is pregnant [1,2]. 

AA is a rare and heterogeneous disease, with an incidence in European countries of approximately two cases per million per year [4]. It is defined as pancytopenia with hypocellular bone marrow in the absence of abnormal infiltrates or marrow fibrosis. Most cases are idiopathic, with a biphasic age distribution between 10-25 years and over 60 years. Some recent studies suggest that acquired AA is caused by a decrease in hematopoietic stem cells associated with an autoimmune etiology [5,6]. Determining the precise etiology of AA is both challenging and essential for effective management, particularly when distinguishing between acquired and inherited forms of the disease [5].

The etiology of our patient’s acquired AA remains unclear, but several hypotheses can be considered. Given the patient’s family history of PNH, an autoimmune response could plausibly explain the bone marrow failure. Although no direct exposure to medications or toxic substances was identified, the possibility of adverse effects from prednisolone or other immunosuppressive agents cannot be excluded. Additionally, despite the absence of suggestive symptoms, an asymptomatic viral infection cannot be ruled out.

At the initial presentation of AA, many patients experience symptoms such as fatigue, weakness, pallor, and headaches due to anemia. Additionally, severe thrombocytopenia often leads to petechiae on the skin and mucous membranes, epistaxis, and gum bleeding. Fever and infections are also common, stemming from low white blood cell counts and neutropenia. However, some AA cases are detected early through routine laboratory tests before any physical symptoms become evident [5,7]. 

The initial diagnostic evaluation aims to differentiate AA from various other causes of cytopenias. This can be particularly challenging, as AA shares overlapping features with other immune cytopenias (both presenting with pancytopenia and hypoplastic bone marrow), paroxysmal nocturnal hemoglobinuria (both displaying pancytopenia), as well as myelodysplastic syndrome and inherited bone marrow failure disorders (both of which may present with cytopenias and hypocellular marrow) [5].

Discrepant results between bone marrow studies conducted at different hospitals have been previously reported [8-10]. Such discrepancies may arise from various factors, including intra-individual biological variation, laboratory errors, the limited resolution of testing methods, the intrinsic nature of cancer cells, cytogenetic biclonality, or hybridization failures [10].

The severity of AA is determined by neutrophil count and, along with age, is a key factor in prognosis and response to immunosuppressive therapy and transplantation. Stem cell transplantation is preferred in young patients, while immunosuppressive therapy is reserved for older patients or those with comorbidities [7]. In this case, despite the severity of the disease, the patient is young with no other comorbidities, which favors her prognosis and response to transplantation, with regular monitoring essential to detect complications such as bleeding and infections, which are the main causes of mortality in AA [7,11].

## Conclusions

Ecchymosis and easy bruising, although often perceived as benign, can be initial indicators of serious underlying conditions such as AA. Accurate diagnosis demands a thorough evaluation that integrates clinical history, laboratory results, and an understanding of the patient’s family background, as these symptoms can emerge from a spectrum of disorders with distinct severities and prognoses. 

In this case, the patient’s family history of PNH guided suspicions toward hematologic abnormalities, yet the diagnostic pathway proved complex. Close correlation between clinical findings and laboratory data is essential, and prompt referral to a hematologist facilitates early diagnosis and effective management. 

Identifying AA at an early stage is particularly crucial for patients without comorbidities since stem cell transplantation offers a favorable outlook. Ultimately, a tailored, integrated approach to both diagnosis and treatment enhances outcomes for patients presenting with unexplained bruising.
